# Supplementary material for: Are carbon emissions trading and green financial instruments synergistic? -Comprehensive quantitative research based on content analysis
Source: PLoS One. 2024 Mar 7;19(3):e0298601. doi: 10.1371/journal.pone.0298601 (PMC10919601; doi:10.1371/journal.pone.0298601)
Supplement: S1 Data — (ZIP) [file pone.0298601.s001.zip › Data/地级市Z_F工作报告.zip!/╕ú╜¿/─■╡┬╩╨/└┤╫╘╣1⁄2╓┌║┼í╛┬φ┐╦╩2╛▌═°í┐_2017.docx]

各位代表：

　　现在，我代表宁德市人民政府向大会作政府工作报告，请予审议，并请各位政协委员和列席人员提出意见。

　　一、2016年和过去五年的工作回顾

　　2016年，在省委、省政府和市委的正确领导下，我市各级政府认真贯彻党的十八大和十八届三中、四中、五中、六中全会精神，学习贯彻习近平总书记系列重要讲话精神，积极适应新常态，认真落实新理念，扎实推进供给侧结构性改革，深入实施环三都澳区域发展战略，加快建设“六新大宁德”，经济社会发展保持总体平稳态势，实现了“十三五”良好开局。预计全市地区生产总值1615亿元、增长7.6%；公共财政总收入155.09亿元、增长5.2%，地方公共财政收入100.97亿元、下降3.3%；固定资产投资1258亿元、与上年持平；出口总值210亿元、下降13.4%；实际利用外资2.31亿美元、增长10%；社会消费品零售总额512亿元、增长10%；居民消费价格总水平上涨1.5%；城镇居民人均可支配收入28240元、增长8.5%；农民人均可支配收入13500元、增长9.0%。

　　一年来的主要工作和成效是：

　　（一）转型升级扎实推进，产业结构逐步优化。工业转型效果显现。实现规模以上工业增加值703亿元、增长8.3%，产值亿元以上企业543家、百亿元以上企业5家。冶金新材料、锂电新能源等新兴产业快速发展，实现增加值275亿元、增长30%，对规模以上工业增长贡献率达120%。高新技术企业29家，新增省级企业技术中心3家、省级众创空间5家、发明专利授权182件。制定全市传统产业转型升级实施方案，分片区、分产业协调推进。实施126项省、市重点技改项目，淘汰落后钢铁产能115万吨。三祥新材成功上市。安波电机获省政府质量奖。现代服务业加快培育。实现服务业增加值525亿元、增长9.0%。旅游产业发展迅速，全市接待游客2168万人次、旅游总收入185亿元，分别增长18%和23%。成功举办第六届宁德世界地质公园文化旅游节。屏南入选首批国家全域旅游示范区创建名单，周宁、福鼎入选省全域旅游试点县。白水洋·鸳鸯溪景区成为首批国家绿色旅游示范基地。三都岛获评中国“十大美丽海岛”。集装箱运输业加快发展，外贸集装箱宁德-厦门内支线开通，完成集装箱吞吐量7万标箱、增长24.3%，港口吞吐量2700万吨、增长6.2%。电子商务较快发展，完成交易额210亿元、增长40%。金融机构各项贷款余额1493.69亿元、增长6.6%。房地产去库存有效推进，商品房销售面积200万平方米、增长15%。现代农业稳步发展。实现农林牧渔业总产值495亿元、增长4.4%。粮食产量61.93万吨，地方粮食储备15万吨。茶叶、水产、食用菌等特色产业加快发展。新增设施农业面积1.05万亩、省级农民合作社示范社11家、省级家庭农场示范场19家、市级以上休闲农业示范点14个。“两权”确权登记颁证工作有序推进，流转土地68.61万亩。古田列入全国“两权”抵押贷款试点县，屏南列入全国农村承包土地的经营权抵押贷款试点县。

　　（二）“五个一批”滚动实施，项目投资有效接续。新增“五个一批”项目412个，总投资3375亿元。实施市级在建重点项目280个，完成投资660亿元，其中年度投资额超10亿元项目10个。列入省行动计划在建重大项目127个，完成投资228亿元。千亿招商计划顺利实现，对接签约项目155个，总投资1175亿元。宁德核电4#机组、新能源二期、青拓不锈钢冷轧及深加工配套、220千伏北洋输变电等一批项目建成投产，中铝铜冶炼、新能源三期、杉杉负极材料、周宁抽水蓄能电站等一批重大项目开工建设，霞浦核电、中铝铜板带箔等项目前期工作扎实推进。福寿高速、宁连高速全线通车，沙埕湾跨海公路通道工程工可获批。衢宁铁路站前工程全线开工建设。湾坞作业区6#7#泊位投入使用。推出两批20个补短板投资工程包，总投资69.28亿元。中心城市东区污水处理厂、福鼎城市道路生活垃圾综合处理项目入选全国第三批PPP示范项目。

　　（三）城乡建设步伐加快，人居环境呈现新貌。中心城区基础设施不断完善，闽东路中段、天山东路一期实现通车，金漳路、漳湾疏港公路、福宁路三期等一批道路加快建设。投放新能源公交车98辆，新增公共停车场7个、停车泊位539个。出租车行业改革不断深化，网约出租车管理逐步规范。建成兰溪公园、环东湖慢行道一期等一批园林绿化项目，新增绿地64万平方米。新建改建供水管道10.6公里、雨污管网42公里。启动建设北区污水处理厂二期工程、第三水厂扩建工程等项目。建成投用一批防洪排涝工程。新型城镇化建设步伐加快，古田列入全国第三批新型城镇化综合试点名单，蕉城赤溪镇列入全国第四批美丽宜居小镇示范名单，福安廉村、蕉城邑坂村、周宁陈峭村、柘荣上黄柏村、古田凤竹村和林峰村列入全国第三批和第四批美丽宜居村庄示范名单。蕉城三都澳大黄鱼小镇、霞浦三沙光影小镇、屏南药膳小镇入选第一批省级特色小镇创建名单。赛岐、太姥山两个“小城市”培育试点三年行动计划获批，全市29个省市小城镇综合改革试点镇实施项目499个。农村环境卫生综合治理扎实开展，改水改厕试点工作成效显著,142个“千村整治、百村示范”美丽乡村加快建设。“两违”综合治理持续推进，拆除违建面积453.72万平方米。

　　（四）改革开放力度加大，关键领域取得突破。创建国家军民融合创新示范区《总体方案》正式呈报国务院、中央军委。宁德军民融合深度发展产业园被工信部列为国家新型工业化产业示范基地，华东地区（福建）船舶综合保障动员中心获批设立。新型护卫舰宁德舰加入人民海军战斗序列。宁德港口岸获准扩大开放三沙港区、临时开放漳湾8#9#码头。中铝矿业（宁德）保税仓库投入使用。医改工作扎实推进，在全省率先全面启动分级诊疗试点工作。组建市医疗保障管理局和市医疗保障基金管理中心，全市医保管理体制实现“三保合一”，城乡居民大病保险全面启动。全面两孩政策得到落实，市县两级妇幼保健与计生技术服务机构完成整合。柘荣被确定为第二批全国健康促进县项目试点地区。行政审批制度改革持续推进，调整规范市级前置审批和中介服务项目280项，市县乡三级实现权责清单全覆盖。“双随机一公开”监管机制有效推行。市公共资源交易中心平台挂牌成立。“五证合一、一照一码”登记制度改革稳步推进，新增市场主体3.63万户、增长30.36%。在全省率先实现市、县不动产统一登记全覆盖。财税体制改革扎实推进，全口径预算编制工作深入实施，“营改增”试点全面推开，政府债务管理不断规范，政府购买服务领域逐步扩大。公务用车制度改革有序推进。居民用水阶梯价格制度全面推行，一般工商业用电同网同价政策正式实施。对外开放与区域合作富有成效，新批外商直接投资和增资项目11个。第七届海峡两岸电机电器博览会和第八届海峡论坛·陈靖姑文化节成功举办，区域协作持续拓展。

　　（五）环境整治成效明显，生态质量保持优良。屏南、寿宁、周宁、柘荣纳入国家重点生态功能区，柘荣获得“国家生态县”称号。全市陆域、海域生态红线以及城市（县城）周边永久基本农田完成划定。在全省率先开展领导干部自然资源资产离任审计试点工作。生态环保目标责任书任务得到有效落实，水、大气、土壤污染防治扎实推进。实施水污染防治项目147个、安全生态水系建设项目10个，重点流域水质功能达标率100%。建成投用乡镇污水集中处理设施15个、垃圾中转设施16个。大唐火电1#2#4#机组完成超低排放改造，燃煤锅炉、黄标车淘汰任务超额完成，空气质量达标天数比例98.6%。危废监管暨规范化管理“达标升级”试点通过考核，市危废综合处置中心项目启动建设，福鼎龙安热电厂、固废处置中心建成试产。植树造林15.67万亩，建设沿海基干林带23.3万亩，治理水土流失21.33万亩。

　　（六）民生福祉不断增进，社会事业长足进步。精准脱贫持续推进，习近平总书记视频连线福鼎赤溪村，肯定我市扶贫脱贫成效。全市实施精准帮扶项目7000多个、投入资金20多亿元，完成造福工程搬迁6560户2.58万人，实现脱贫3.8万人。保障体系逐步完善，全市民生支出196.11亿元、增长5.9%，占公共财政支出72.9%。城镇新增就业3.14万人、登记失业率2.79%，农村劳动力新转移就业4.12万人。27个为民办实事项目基本完成。基层人社服务平台建设走在全省前列，发放城乡居民养老金4.95亿元，1895名高校毕业生实现自主创业。福利和慈善事业持续发展，市社会福利中心投入运营，新建农村幸福院42个。最低生活保障制度惠及10.6万人。新开工保障性安居工程7478套，基本建成7760套，累计配租配售保障性住房2.59万套，完成农村危房改造2000户。公共服务稳步提升，实施中小学扩容工程12项、校安工程20项、公办幼儿园16所。屏南、周宁通过义务教育发展基本均衡县国家评估认定。宁德师范学院通过教育部本科教学工作合格评估。宁德职业技术学院入选省示范性高职院校培养单位名单。文化惠民活动常态推进，文艺精品创作富有成效，《山哈魂》获全国少数民族文艺会演银奖。新增中国传统村落32个。群众性精神文明创建活动深入开展，“学习大军”民间志愿组织入选“全国百佳”。第十六届省运会筹办和备战工作扎实推进，市首届“魅力农体”展示大会顺利举办。“平安宁德”建设不断深化，反恐维稳管控和治安重点防控措施有力，各类刑事犯罪得到严厉打击，群众安全感达94.56%。圆满完成G20杭州峰会安保任务，福鼎贯岭公安检查站被公安部授予集体一等功，“七五”普法启动实施，宗教领域总体和谐稳定，信访态势平稳可控，安全生产形势保持稳定，食品药品安全保障有力。防抗台风及灾后重建工作扎实有效。连续四届被评为“全国双拥模范城”，国防后备力量、民兵预备役建设、海防、人防和打私工作进一步提升。同时，工、青、妇、计生协、老体协、科协、统计、老区、老龄、残联、移民、人事编制、民族、审计、档案、地方志、气象、防震、外事、侨务、对台等工作也都取得新进展。

　　（七）自身建设持续加强，工作作风得到改进。扎实开展“两学一做”学习教育，认真履行全面从严治党主体责任，加强党风廉政建设和反腐败工作，持续反对“四风”，严格落实中央八项规定精神。坚持依宪施政、依法行政，修订完善《宁德市人民政府工作规则》，出台《宁德市人民政府拟定法规草案和制定政府规章程序规定》，科学决策、民主决策、依法决策水平进一步提高。自觉接受人大及其常委会监督并报告工作，依法执行人大及其常委会决议、决定，积极配合开展执法检查、代表视察、专项调研、工作评议和专题询问，办理市人大代表建议259件。支持政协履行政治协商、民主监督、参政议政职能，定期向政协通报情况，重视委员调研协商成果，办理市政协提案212件。主动接受司法、社会和舆论监督。坚持“四下基层”，推行“马上就办”，机关效能建设和绩效管理持续加强。

　　各位代表，随着2016年工作的收官，本届政府任期目标任务已顺利实现。五年来，我们在抢抓机遇中加快发展，在应对挑战中克难奋进，谱写了宁德发展新篇章。

　　——这是宁德综合实力跃升晋位的五年。全市地区生产总值先后突破千亿元和1500亿元，年均增长10.4%，高于全省平均增幅。人均地区生产总值突破5万元。规模以上工业总产值突破3000亿元。公共财政总收入和地方公共财政收入先后突破百亿元，均前移至全省第七位，区域经济竞争力进一步增强。

　　——这是宁德产业发展转型提升的五年。深入实施产业转型升级和《中国制造2025》行动计划，着力做大增量、优化存量。特色现代农业产业体系基本形成，农林牧渔业总产值年均增长5.3%。地理标志驰名商标连续四年居全国设区市首位。工业化进程加快，形成7个百亿产业集群，冶金新材料产业产值近千亿元，锂电新能源产业产值突破200亿元。民营企业上市实现零的突破。第三产业稳步发展，游客接待量、旅游综合收入实现翻番，新增3A级以上景区13个，金融机构从18家增加到60家，在全省率先实现村镇银行县域全覆盖，电商、物流等业态发展迅速，新亮点不断涌现。

　　——这是宁德城乡面貌显著变化的五年。全市常住人口城镇化率和户籍人口城镇化率分别达55%和35.32%。中心城市主城区面积从22.8平方公里拓展到34.2平方公里，人均道路面积12.35平方米，人均公园绿地面积15.2平方米，成功创建国家园林城市。森林覆盖率67.18%，森林蓄积量4422.26万立方米。省级生态县（市、区）创建实现全覆盖。建成美丽乡村示范村40个，打造美丽乡村景观带19条，国家级传统村落数量居全省第一。

　　——这是宁德基础设施取得突破的五年。固定资产投资累计完成5172亿元，是前五年的3.5倍，能源、交通、市政、农业、通信基础设施加快建设。电力装机总容量新增945万千瓦，在全省率先跨入“核电时代”。新增高速公路通车里程257.2公里，实现“县县通高速”。新增铁路通车里程19.8公里、普通公路通车里程1856公里、港口吞吐能力732万吨。

　　——这是宁德民生保障持续改善的五年。扶贫开发“宁德模式”得到国家层面肯定，累计脱贫16万人。造福工程搬迁2.95万户11.8万人。建成保障性住房2.8万套。全市财政民生支出每年都占公共财政总支出66%以上。城乡居民人均可支配收入分别增加10527元、5681元，年均分别增长9.8%、11.5%。“双高普九”提前实现，义务教育发展基本均衡实现全覆盖，文化、卫生、体育等公共服务体系加快完善。社会保障制度不断健全，物价水平保持稳定，社会治理水平逐年提升，综治考评成绩居全省前列，进入全省文明城市行列。

　　各位代表，回顾五年历程，成绩来之不易。这是中央和省委、省政府正确领导、关心支持的结果，是在市委坚强领导下全市干部群众团结一心、奋力拼搏的结果，是市人大、市政协加强监督、鼎力支持的结果。在此，我代表市人民政府，向全市人民，向人大代表、政协委员、各民主党派、工商联、各人民团体、无党派人士、离退休老同志和社会各界人士，向中央及省属驻宁机构、驻宁部队、武警官兵、公安民警，表示衷心的感谢和崇高的敬意！

　　在肯定成绩的同时，我们也清醒认识到，发展中还存在不少困难和问题，主要表现在：经济下行压力大，实体经济困难加剧，防范和化解金融风险任务依然艰巨；传统产业转型步伐偏慢，企业自主创新能力亟需加强，环境刚性约束趋紧，节能减排压力较大；城乡规划、建设、管理水平有待提升，中心城市辐射带动力不强，城镇化水平不高；扶贫对象仍然较多，因病因灾致贫返贫现象时有发生，脱贫攻坚任务繁重紧迫；教育、卫生等基本公共服务水平与群众期望还有较大差距，社会治安、安全生产等领域还存在一些隐患；一些干部担当意识、服务意识不强，依法行政水平还要进一步提高。对此，我们一定要高度重视，采取有力措施加以解决。

　　二、今后五年发展的总体要求和主要目标任务

　　今后五年，是全面建成小康社会的决胜期，是全面深化改革的攻坚期，也是我市科学发展、加快发展的关键期。综观国内外形势，世界经济在深度调整中曲折复苏，新一轮科技革命和产业变革正在兴起，新常态下，我们既有下行压力，又不乏发展机遇。经过十多年高速增长，我市经济发展跃上新台阶，产业发展初具规模，交通、市政基础设施不断完善，一批大项目好项目积蓄了发展后劲，环三都澳区域发展集中度逐步提升，以港兴城、产城联动格局正在形成，特别是中央和省里支持宁德加快发展的利好政策叠加，更加凸显了我市海洋、生态、军民融合等优势。我们要坚定信心、振奋精神，滴水穿石、久久为功，把各项事业继续向前推进。

　　今后五年，政府工作的总体要求是：高举中国特色社会主义伟大旗帜，以马克思列宁主义、毛泽东思想、邓小平理论、“三个代表”重要思想、科学发展观为指导，深入学习贯彻习近平总书记系列重要讲话精神及对宁德的重要指示批示，围绕统筹推进“五位一体”总体布局和协调推进“四个全面”战略布局，牢固树立和贯彻落实新发展理念，主动融入“再上新台阶、建设新福建”大局，按照省市党代会的部署，紧紧扭住发展第一要务，牢牢把握转型升级、提质增效这一主线，深入实施环三都澳区域发展战略，努力建设机制活、产业优、百姓富、生态美的新宁德，确保如期全面建成小康社会。

　　今后五年，我们的奋斗目标是：综合实力更加强大。全面完成“十三五”规划确定的目标任务，地区生产总值超2500亿元，人均地区生产总值达8.5万元，向全省中游水平迈进。培育若干个千亿产业集群和百亿企业，形成以主导产业和现代服务业为主体、特色现代农业为基础的产业发展体系。改革开放更加深化。重点领域和关键环节改革取得实质性进展，政府职能转变步伐加快，创新型宁德建设深入推进，环三区域开放开发、生态文明建设、军民融合深度发展等事关长远的重大工作取得突破。城乡发展更加协调。城市规划、建设、管理水平进一步提高，新型城镇化加快推进，中心城区扩容提质,特色县城（小镇）加速打造，“小城市”试点培育、“美丽乡村”建设显著提升，港城一体、产城融合、城乡统筹格局加快形成。生态环境更加优美。主动融入国家生态文明试验区建设，重点生态功能区得到有效保护和管理，环境突出问题得到有效治理，“绿水青山就是金山银山”意识深入人心，生态环境质量保持全省前列。人民生活更加幸福。居民人均可支配收入超3.2万元，现行标准下贫困人口全部脱贫，基本公共服务均等化水平不断提高，广大群众获得感、幸福感进一步增强。

　　今后五年的中心任务，归结起来就是：“开发三都澳、建设新宁德”。“开发三都澳”，是习近平总书记在宁德工作期间提出的宏伟蓝图，是闽东人民梦寐以求的夙愿。环三都澳区域发展战略作为福建省发展战略的重要组成部分，通过近10年的实施建设，具备加快开发的基础条件，迎来加快发展的新机遇。我们要坚持一张蓝图干到底，把握国家、省里支持创建军民融合创新示范区的机遇，深入实施环三都澳区域发展战略，有序推进“一港五区”布局建设，着力打造“一核六放射”疏港大通道，进一步整合和发挥“临海”资源优势，完善和凸显“环海”布局构架，港城联动、陆海统筹、连片开发、环湾发展，逐步向“跨海”开放发展跃升，成为福建新的增长极。要坚持以港兴城，以港口开发带动城市加快发展，以城市建设推进港口腹地延伸；要坚持产城融合，以城市为基础，以产业为支撑，拓展生产生活空间，加快城市更新、功能完善；要依托区域资源禀赋和产业基础，坚持差异化发展，念好“山海经”，搞好“大合唱”，推动沿海蓝色经济带加快开发、山区绿色发展带多点支撑，进一步振兴县域经济。“建设新宁德”，是“再上新台阶、建设新福建”的必然要求，是实施环三都澳区域发展战略的奋斗目标，我们要坚持改革为先、创新为重、人才为要，多上好项目、多抱“金娃娃”，加快发展新经济、培育新动能，切实增强综合竞争力；要把握政策机遇，积极探索、奋力攻坚，在扶贫开发、产业转型、军民融合、新型城镇化和生态文明建设等方面创新突破，推动宁德在新的起点上实现更高水平发展。

　　“开发三都澳、建设新宁德”，是宁德发展的主旋律，各级各部门必须围绕这一坐标定位，主动融入、提升站位、务实举措，大力弘扬“滴水穿石”精神，切实强化“弱鸟先飞”意识、“行动至上”作风和“四下基层”制度，一步一个脚印把美好蓝图变成现实。

　　三、扎实做好2017年政府工作

　　今年是实施“十三五”规划的重要一年，是推进供给侧结构性改革的深化之年。我们要坚持稳中求进工作总基调，积极适应经济发展新常态，围绕“开发三都澳、建设新宁德”的目标，深化创新驱动和供给侧结构性改革，扎实开展“一转三抓比贡献”活动，全面做好稳增长、促改革、调结构、惠民生、防风险各项工作，推进“三去一降一补”五大任务取得实质性进展，努力提高发展的质量和效益，推动经济社会平稳健康发展。

　　经济社会发展的主要预期目标是：全市地区生产总值增长8%、力争8.5%；公共财政总收入和地方公共财政收入均增长5%左右；固定资产投资增长10%；出口总值增幅不低于全省平均水平，实际利用外资增长6%；社会消费品零售总额增长10.5%，居民消费价格总水平涨幅控制在3%以内；城镇居民人均可支配收入增长8.5%，农民人均可支配收入增长9%；城镇登记失业率控制在4%以内；完成单位生产总值能耗、化学需氧量、二氧化硫、氨氮、氮氧化物等年度节能减排任务。

　　实现上述目标，重点要抓好八个方面工作：

　　（一）加快产业转型升级

　　着力振兴实体经济。加快培育新动能。坚持抓龙头、铸链条、建集群，推进青拓系列、宏旺二期、克虏伯、海利等项目建设，打造冶金新材料千亿产业集群。推进新能源三期、卓高隔离膜、杉杉负极材料、厦钨正极材料等项目建设，打造锂电新能源500亿产业集聚区。推进霞浦核电、周宁抽水蓄能电站、宁德核电5#6#机组及一批风电项目建设，建设东南沿海重要清洁能源基地。推进中铝铜冶炼、铜板带箔等项目建设，延伸铜冶炼、加工、贸易全产业链。改造提升传统动能。用好省里扶持我市产业转型升级的政策措施，继续推动合成革、电机电器、食品加工、化油器、船舶修造和医药、医疗保健器材等传统产业转型升级。支持龙头企业增资扩产、兼并重组、延伸链条。实施100项以上重点技改项目，完成投资120亿元。整合提升工业园区。做大做强10个省级工业园区，完善园区基础设施和公共服务配套，引导企业“进区入园”，促进产业集聚发展。

　　培育壮大现代服务业。加快旅游业发展，完善旅游产业管理体制，整合旅游资源，提升景区品质，推动“旅游+”跨界融合。推进屏南、福鼎、周宁全域旅游创建工作。办好第七届宁德世界地质公园文化旅游节，做好迎接二次中期评估准备工作。力争全市游客接待量、旅游总收入分别增长16.5%和21%。大力发展商贸物流，抓紧建设宁德火车站综合客运枢纽站、水陆联运物流中心等项目。支持做大中铝矿业（宁德）保税物流业务，积极申报建设宁德保税物流中心。深化与中远海运等大型物流企业合作。抓好宁德大润发、古田中央名城、福安东百广场等城市综合体建设。改造提升现有专业批发市场。大力发展电子商务，重点推进古田、福安、屏南等省级农村电子商务示范县建设，加快培育电商产业园、创业园，支持京东、淘宝等大型电商平台“宁德馆”规范建设。继续“引银入宁”，支持金融业态、产品和服务创新。促进房地产市场平稳健康发展。

　　　大力发展特色现代农业。实施粮食安全保障工程，落实惠农扶粮政策，确保粮播面积196万亩、产量63万吨。巩固发展茶叶、水产、食用菌、果蔬、林竹、畜禽、中药材等特色产业。提升农产品精深加工和质量安全水平，扩大农业品牌效应。推广“1+N”发展模式，促进一产接二连三。推进农村土地“三权分置”，完成土地承包经营权确权登记颁证。加快现代农业示范园区（基地）建设，鼓励农村土地流转，引导农业适度规模经营。培育新型农业经营主体，新增市级以上农民合作社示范社30家以上、家庭农场示范场20家以上。推广农业“五新”100项以上，支持生态农产品开发，拓展特色农业新领域。充分发挥农机、气象、水文等服务保障作用，提高农业防灾减灾能力。注重发展休闲渔业、远洋渔业，大力发展现代高效设施渔业，推进湾外深水抗风浪网箱养殖基地建设和内湾环保塑胶网箱升级改造，推广陆上工厂化循环水养殖，加快三都澳大黄鱼产业园区建设。

　　（二）积极扩大有效投资

　　突出重点领域投资。实施列入省行动计划的在建重大项目126个、总投资1900亿元，年度计划投资220亿元。实施市级重点项目388个，其中在建项目288个，年度计划投资547亿元，力争开工项目90个、竣工项目70个。持续加大交通、水利、电力、通信等基础设施投资力度。建成4个普通国省道项目，加快建设衢宁铁路及相关配套工程、屏古联络线、沈海复线福鼎贯岭至柘荣段、福安至蕉城漳湾段等项目，开工建设沙埕湾跨海公路通道工程、宁古高速等重大交通项目，加快推进宁德至南平铁路前期工作。建成官昌水库主体工程，继续建设万里安全生态水系、引调水工程、抗旱水源等民生水利项目，力争开工建设上白石水利枢纽工程，加快推进闽东苏区中心城区防洪防潮、军民融合供水保障工程等项目前期工作。建成投产500千伏崇儒输变电项目，加快建设220千伏古田月头山、周宁金钟、蕉城漳湾等输变电项目。

　　　提高招商引资成效。继续实施千亿招商行动计划，坚持以商招商、产业链招商、行业性招商，瞄准世界500强、大型央企国企民企的投资趋向，积极对接市场前景好、投资规模大、带动能力强的大项目、好项目。注重长中短期项目统筹、大中小项目配套、政民外项目齐抓，高质量谋划好“五个一批”项目，形成滚动接续格局。坚持招商与留商并重，加快破解审批、安征迁、融资等难题，优化投资软环境，助推项目落地建设。围绕做大做强冶金新材料、新能源等产业，依托青拓系列、宁德新能源、时代新能源、中铝铜冶炼等龙头企业，争取在产业链关联项目引进上取得新的突破。

　　有效激活民间投资。鼓励和引导社会资本参与基础设施和公共事业项目建设运营,提高公共服务质量和效益。继续实施易地扶贫搬迁、特色小镇、城市公共停车场等一批补短板、惠民生投资工程包。创新投融资体制，拓宽政府性投资项目融资渠道，加快实施39个PPP示范项目，支持有条件企业开展股权和债权融资。鼓励企业上市融资，积极争取各类产业基金、专项建设基金支持。加强产权保护，落实好国家减税降费各项政策。坚决破除民间投资的“玻璃门”和“弹簧门”，充分调动民间资本投资积极性。

　　（三）奋力推进脱贫攻坚

　　坚决完成脱贫攻坚军令状。巩固提升扶贫开发“宁德模式”，紧盯脱贫攻坚节点任务，落实“五个一批”扶贫到户措施，进一步完善“664”精准扶贫工作机制，全年完成减贫3万人以上，确保目前建档立卡的国定标准扶贫对象1.55万人全面脱贫。继续支持省级扶贫开发重点县加快发展，推动屏南国家级扶贫改革试验区建设。加大苏区、老区基点村、少数民族聚居区、海岛、库区等帮扶力度。启动新一轮208个扶贫开发重点村整村推进计划，完成造福工程搬迁1.5万人，建成50户以上集中安置点20个。

　　坚持走开发式扶贫路子。依托区位条件、资源禀赋，引导和帮助贫困户发展设施农业、林下经济、休闲农业、乡村旅游等特色产业项目，让贫困户更多地分享到农业全产业链和价值链增值收益。坚持扶贫先扶智，加强教育扶贫和技能培训，开展“雨露计划”和“春潮行动”培训2.45万人，为贫困户提供良好的就业创业服务，让贫困户有长期稳定的务工收入。落实好小额信贷扶贫政策，满足“有劳力缺资金”的贫困户贷款需求，力争全年发放扶贫小额信贷2亿元。

　　全面落实兜底式扶贫政策。统筹协调农村扶贫政策和低保制度，对贫困对象中完全或部分丧失劳动能力的，由社会保障兜底，实现应保尽保。深入细致做好因病因灾致贫返贫户救助工作，加强分类指导，加大扶持力度，确保应扶尽扶。进一步关心关爱农村留守儿童、留守妇女、留守老人。

　　（四）统筹推进城乡建设

　　　强化城乡规划引领。探索完善市、县空间规划体系，科学规划城乡建设、土地利用、产业发展、生态环保等空间布局，注重“留白、留绿、留旧、留文、留魂”。启动中心城区“多规合一”，完成福鼎“多规合一”试点工作。实施城市综合交通、排水防涝、绿地系统等专项规划，完成各县（市）城市景观风貌、中心城区地下管廊等规划。加强各专项规划与控规衔接，提高建设用地控规覆盖率，实现中心城区建设用地控规全覆盖。

　　做大做强中心城区。坚持“东扩面海、北展南移”，提高城区集聚辐射能力。进一步明确漳湾临港、七都八都、三都岛、滨海新城等组团定位，促进漳湾临港工业园区和新能源产业集聚区等北部新区发展。加快实施井上片区、滨海大道及内侧围填海项目，推动金塔、东兰组团和飞鸾片区集中发展。拓宽改造104国道金涵大桥至漳湾疏港公路，续建福宁北路、南天路、天德路、金马路、学院路等城市交通干道，拓展城市骨架。布局建设停车场、农贸市场等公共配套设施。实施八一五路东段、闽东无线电厂、鹤岭小区等棚改项目。加快推进东区污水处理厂、贵岐山污水处理厂技改扩建、雨污分流改造二期工程，启动东湖塘北港黑臭水体治理、东湖清淤前期工作。动工建设人民广场、环东湖慢道二期、西陂公园二三期等园林绿化景观工程。

　　加快新型城镇化步伐。完成古田国家级和福安、屏南省级新型城镇化综合试点方案编制报批。实施省市小城镇综合改革试点项目350个、计划投资80亿元。加大赛岐、太姥山两个“小城市”试点机构改革力度。加强蕉城、霞浦、屏南省级特色小镇探索创新，因地制宜建设一批工贸、文化、旅游、电商小镇。打造美丽乡村“升级版”，新增4条以上美丽乡村景观带，推进第三轮21个少数民族特色村寨试点建设。建成10个乡镇污水处理设施、20个垃圾中转站，选择12个行政村开展农村垃圾资源化、减量化试点，全面推开改水改厕工作，推动更多城镇基础设施、公共服务向农村延伸覆盖。

　　加强城市精细化管理。加快“智慧宁德”建设，加强物联网、云计算、大数据等新一代信息技术应用，推行“互联网+政务”“互联网+民生”等服务新模式。深化城市建设管理体制改革，进一步理顺中心城区城市管理执法体制，推行县（市、区）一级执法，下移执法重心。完成市、县城市管理机构设置，明确城市管理职责。启动实施新一轮“两违”综合治理三年专项行动。下大气力解决群众反映强烈的交通堵、停车难以及脏乱差等问题。

　　（五）提升开放开发水平

　　推进港口开发。立足环三都澳区域发展，坚持“深水深用、浅水浅用，规划引导、梯度建设”原则，有序推进“一港五区”连片开发。加快建设三都澳深水航道二期工程，续建城澳作业区1#2#、杨岐作业区16#泊位，新建漳湾作业区10#、湾坞作业区1#5#12#13#泊位，建成八尺门作业区3#4#泊位。启动城澳作业区6－15#泊位连片开发前期工作，完善三沙、沙埕、白马港区和漳湾8#9#码头等配套设施。加快306省道漳湾至104国道濂坑段、228国道城澳至罗源界段等疏港公路建设。加强锚地、航道建设、使用和管理，严厉查处违法占用岸线、擅自改变岸线使用性质和功能的行为，持续推进海上无序养殖清理或转移。

　　　扩大对外开放。主动融入“海丝”核心区建设，拓展与“海丝”沿线国家的人文交流、港航互动和经贸合作。借鉴自贸区经验，加快通关一体化改革，推进国际贸易“单一窗口”建设，促进贸易便利化。继续扩大电机电器、水产品等传统产业出口，发展壮大新能源、冶金新材料、生态合成革等出口新增长点。完善外贸公共服务平台，培育一批市级外贸综合服务（试点）企业。深化大黄鱼、茶叶、食用菌国家级出口质量安全示范区建设。支持企业参加境内外专业性重要展会。加强外事侨务工作，密切与海外侨团、旅外乡亲社团、友城联系交往。积极推动福莆宁和平潭、闽东北五市一区等区域互动合作发展。

　　深化对台交流。办好第八届海峡两岸电机电器博览会，加强与台湾工商团体联系，深入对接台湾百大企业、行业龙头和科技型中小企业，密切宁台现代物流、生态旅游、港口交通、精细农业、食品加工等领域协作。推进霞浦台湾水产品集散中心、宁台农业合作示范基地建设，鼓励台湾青年来宁创业就业。举办第九届海峡论坛·陈靖姑文化节，促进圆瑛、甘国宝、马仙等特色文化和教育、科技、卫生、体育方面交流。

　　（六）激发改革发展活力

　　推进军民融合发展。继续跟踪对接，争取国家尽快批准我市设立军民融合创新示范区。完善示范区总体规划、专项规划，加强军民融合产业、军地共办社会事业以及交通基础设施等项目建设。对接十大军工企业，策划引进一批军工项目。发挥国家新型工业化产业示范基地和华东地区（福建）船舶综合保障动员中心等平台作用，推动船舶修造、电机电器、冶金新材料、锂电新能源等企业研发、生产军民两用产品。深化与国防科技大学、解放军装备学院合作，扎实推进技术转化和创新平台建设。加强与军队沟通对接，争取在扩大三都澳水域开放上取得新进展。做好双拥创模工作，争创“省级双拥模范城”五连冠。

　　深化重点领域改革。继续实施“放管服”改革，严格清单动态管理，扩大网上审批、并联审批。全面建立“两单两库一细则一平台”，强化事中事后“双随机一公开”监管。认真落实“五证合一”，探索推进“多证合一”，加快工商登记全程电子化步伐，推进企业登记注册、注销便利化改革。深化国有企业改革, 加快国有资产整合，提升企业自营能力和造血功能。进一步推进政府预决算公开，加强预算绩效管理，逐步扩大政府购买服务范围。推进分级诊疗全覆盖，完善公立医院内部运行管理机制，加快公立医院和基层医疗机构综合改革。实施同心全科医生特岗人才计划，充实基层卫生人才队伍。鼓励和支持社会力量办医。深化机关事业单位养老保险制度改革。推进售电侧改革试点和农业综合水价、终端气价阶梯价格改革。

　　构建人才支撑体系。加快创新型宁德建设，深化人才发展体制机制改革，着力培养、引进和用好各类人才，致力打造企事业人才洼地。持续深化与北大、清华等国内著名高校的合作。建设市级公共实训基地，支持企业建设国家级或省级工程技术研究中心、重点实验室和院士专家工作站、博士后科研工作站，促进高层次创新人才集聚。建立健全人才评价认定、流动、激励和使用机制，营造人尽其才、才尽其用的良好氛围。

　　（七）巩固拓展生态优势

　　　加强生态环境保护。主动融入生态文明试验区建设，对接落实年度改革试验任务，打造生态保护绿色发展高地。严格落实环保“党政同责”、部门“一岗双责”，加快构建绿色发展指标体系，开展领导干部自然资源资产离任审计，建立生态环境损害责任终身追究制。加强屏南、寿宁、周宁、柘荣4个国家重点生态功能区生态保护和修复，实行产业准入负面清单，合理调控工业化城镇化开发内容和边界，出台重点流域生态补偿管理办法，提高生态产品供给能力。建立生态红线管控制度，实施湿地面积总量管理，加强海岸线分类分级保护，严格自然生态空间征（占）用管理。建立健全企业环境信用评价制度。造林绿化7.96万亩，综合治理水土流失15万亩。

　　深化环境综合治理。全面落实“河长制”，强化水污染防治工作，促进8条小流域水体达标与水质提升，推进工业、畜禽养殖、近岸海域污染和城市黑臭水体等专项整治。抓好火电、冶金等重点行业大气综合治理，统筹整治燃煤锅炉、建筑扬尘和餐饮油烟，加快淘汰落后钢铁产能，基本淘汰黄标车，实现大气污染防治计划目标。加快落实“土十条”，推动敖江流域矿山生态环境恢复治理，加快饰面石材加工企业转产转业。建设或建成全市危废综合处置中心、污泥处置中心、餐厨垃圾处理厂。完善网格化环保监管体系，持续开展环保专项执法行动，强化重金属、危险废物、核与辐射等环境监管。

　　推进绿色低碳发展。大力发展绿色产业，严控“两高”行业，实行能源和水资源消耗、建设用地等总量和强度双控。实施节能重点项目10个以上，加快推进宁德新能源、时代新能源、榕屏化工等企业循环经济试点工作。落实各类开发区、工业区污水全收集全处理，加强重点行业污染集中治理。全面实施排污权有偿使用和交易工作，推行合同能源管理、环境污染第三方治理。强化水资源“三条红线”管理，推广节水技术和产品。倡导文明、节约、绿色、低碳的生活理念和消费方式。

　　（八）切实保障和改善民生

　　加大社会保障力度。建立经济发展和扩大就业联动机制，完善就业创业服务体系，多渠道促进高校毕业生、就业困难人员、农民工和失业人员实现就业创业，力争全市新增城镇就业2.8万人、失业人员再就业1万人。实施“全民参保登记计划”。深化无欠薪项目部信息化建设，积极构建和谐劳动关系。建成9个县（市、区）就业和社会保障公共服务中心。新开工保障性安居工程5188套，基本建成保障性住房和棚改安置房1269套。积极发展养老服务业和老龄产业，鼓励发展新型家政服务、社区服务，加快建设社区老年人日间照料中心。推进健康宁德建设，加快实施市中医院医养结合、福鼎市医院百胜新院区等项目，启动古田、柘荣、福安等县（市）精神病医院建设。重视妇幼保健、疾病预防控制、卫生应急处置等工作。推进全面两孩政策落实，促进人口资源均衡发展。

　　推进文化强市建设。培育和践行社会主义核心价值观，全面落实宣传舆论导向等“六大行动计划”，大力弘扬周炳耀等先进典型事迹。认真筹办第十六届省运会和第十届省老健会，全面完成比赛场馆建设，广泛开展“创文明城、迎省运会”系列活动，提高城市文明程度和市民文明素质。开工建设市博物馆、档案馆、科技馆和青少年宫，加快建设一批基层综合性文化服务中心，推动基本公共文化服务标准化、均等化。支持“学习大军”品牌建设，促进报纸、广播电视等传统媒体和网络、微信等新兴媒体深度融合，繁荣清朗网络空间。落实“十三五”特色文化发展专项规划，实施文化精品创作工程，打造“一县一品”文化品牌。推进省级畲族文化生态保护示范点建设，筹建市畲族文化生态园。实施闽东苏区红色文化“六个一批”工程，推进蔡威红色文化“五个一”精品工程，提升寿宁下党等一批红色旅游品牌基地。积极推动文化与旅游、科技、影视、展会、互联网等领域的多元融合，打造文化产业升级版。完成第二轮志书修编出版。

　　坚持教育优先发展。全面加快义务教育薄弱学校改造，实施校安工程14项，新建10个公办幼儿园，计划新增学位8200个。推进普惠性民办幼儿园建设。落实中小学教学质量提升三年行动计划，创建77所义务教育管理标准化学校。加强中考和中职招生制度改革，实现高中阶段普职招生比大体相当。深化产教融合、校企合作，打造“二元制”现代职业教育。加快宁德师院三期和宁德职业技术学院、闽东卫校新校区建设。加强教师队伍建设，实施乡村教师支持计划。重视发展继续教育、民族教育、特殊教育、老年教育。

　　维护社会安定稳定。深化“平安宁德”建设，完善立体化治安防控体系，全面加强消防、禁毒、反走私、反恐、交通管理等工作。强化社会治安综合治理创新，完善多元化矛盾纠纷化解机制，落实信访工作责任，创新信访工作机制。严格落实安全生产责任制，加强重点行业领域安全生产专项整治，确保人民群众生命财产安全。深化“餐桌污染”治理和药品安全监管，保障人民群众饮食用药安全。持续防范和化解信贷风险，加强诚信体系建设，优化金融生态环境。充分发挥工会、共青团、妇联等人民团体的作用，重视做好国防动员、后备力量建设、民族宗教、防震、气象、统计、档案、老龄、残疾人和关心下一代等工作。

　　四、加强政府自身建设

　　各位代表，新常态要有新状态，新起点要有新作为。我们一定要提振精气神，凝聚正能量，奋力求作为，切实增强发展紧迫感和责任感，切实加强政府自身建设，切实提高执行力、创新力和公信力，不断开创改革发展新局面。

　　对党忠诚，严守纪律规矩。牢固树立“四个意识”特别是核心意识、看齐意识，坚决维护以习近平同志为核心的党中央权威，始终在思想上、政治上、行动上同以习近平同志为核心的党中央保持高度一致。坚决落实全面从严治党要求，把纪律和规矩挺在前面，切实加强党风廉政建设和反腐败工作。自觉践行党章和党内政治生活准则、廉洁自律准则、纪律处分条例、问责条例、党内监督条例等，持之以恒落实中央八项规定精神，以严实作风应对新挑战、增创新业绩。

　　加强学习，提升履职能力。强化思想教育和理论武装，加强党性修养和锻炼，坚定中国特色社会主义道路自信、理论自信、制度自信和文化自信。增强“本领恐慌”意识，有针对性地开展经济管理、科技创新、文化建设、法治建设、社会治理、生态文明等方面培训，优化知识结构，增强领导能力，提高决策水平。加强干部队伍专业化建设，提升各级干部的专业思维、专业素养和专业能力。坚持理论学习与实践历练并举，集中选派干部到改革、发展、稳定、民生一线挂职锻炼。

　　依法行政，规范权力运行。坚持在党的领导下、在法治轨道上开展工作，依法全面履行政府职能，健全科学民主决策机制，完善依法行政制度体系，严格规范公正文明执法，提高运用法治思维和法治方式的能力。依法规范施政行为，坚持法无授权不可为的理念，切实做到权为民所用、利为民所谋。依法接受人大及其常委会的法律监督和工作监督，主动接受政协民主监督，认真听取各民主党派、工商联和无党派人士意见，提高人大代表建议、意见和政协提案办理质量。加大行政监察和审计监督力度，全面推进政务公开工作。

　　开拓创新，展现担当作为。坚决破除思维定势、工作惯性和路径依赖，在面对难事要事上勇于担当、直面矛盾、破解难题，在服务发展与振兴实体经济上大胆创新、锐意进取、展现作为。坚持“四下基层”，强化“马上就办”，加强和改进绩效管理，加大问责力度，整肃懒政怠政行为，着力提高政府执行力。认真落实“五个一批”举措，健全差异化考评体系，探索建立容错纠错机制，为呕心沥血、不谋私利、勇于攻坚的干部撑腰鼓劲，形成人人想干事、敢干事的环境和氛围。

　　各位代表，“开发三都澳、建设新宁德”目标催人奋进。让我们紧密团结在以习近平同志为核心的党中央周围，在省委、省政府和市委的坚强领导下，同心同德，励精图治，为全面建成小康社会、实现第一个百年目标而努力奋斗！
